# Normal olfaction range of Rasht residents using a new test designed for the region

**Published:** 2010

**Authors:** Hooshang Gerami, Shadman Nemati, Rahmatollah Banan, Rezvan Rouhi

**Affiliations:** a Otolaryngology Head and Neck Surgery Department and Research Center, Guilan University of Medical Sciences, Faculty of Medicine, Rasht, Iran; b Resident of Otolaryngology Head and Neck Surgery, Guilan University of Medical Sciences, Faculty of Medicine, Rasht, Iran

Disorders of sensing smell and taste can present diagnostic and therapeutic challenges for the otolaryngologists,^1^ especially for forensic problems and especially in Islamic countries- the importance of olfactory sensation in Islamic laws is as high as whole body of a human being.

There are many methods for qualitative and quantitative evaluation of olfactory sensation in the world.^2^ Discrimination and detecting of odors is largely cultural and therefore learnt 3; hence, it is not surprising if the smell tests which had been designed and applied in other cultures and countries would have some restrictions in being applied in another country.^3^–^5^

Unfortunately there isn’t any locally designed test currently used in Iran. Also, the current tests are difficult to interpret and are usually expensive to run.

In order to develop a standard smell test for Iranians and determination of the normal range of olfactory sense in this region, a research composed of two stages was conducted; to find the most popular odorous items in the region, fifty six well known materials were presented to two hundred, 15- 60 years old, normal residents of Rasht, Guilan province, Iran. Sixteen materials with the highest scores were selected in this stage: gasoline, alcohol, tea, rice, soap, cinnamon, origan, garlic, onion, washing powder, mint, rose water, lemon juice, olive oil, vinegar, and menthol. Fifteen of these were the olfactory nerve stimulators, and one (menthol salicylate) was the trigeminal nerve stimulator; this is unlike other tests that use ammonia as the trigeminal nerve stimulator, because ammonia has tang and unpleasant smell and in high doses may damage the olfactory system.

Then, these 16 materials were presented in identical containers to 150 normal citizens (77 women, 73 men) at 2 centimeter distance from their noses, for 3 seconds. Each nostril was tested separately; therefore the total score for two nostril and 16 materials was 32 for each person. The subjects would choose an answer in a 4 choice questionnaire; so, malingering individuals can be identified (someone who had less than 25% of total score). Thirty of the subjects were re- tested with the designed sniff box for evaluation of reproducibility of the test.

Considering 97 percentile, the normal range of olfactory score for 15- 60 years old residents of Rasht was 28- 32. The mean scores in different age and sex groups are presented in table 1. Test re test reliability was more than 96%.

**Table 1:** Mean of olfactory scores in different age and sex groups

Age groups (years)	Male		Female	
	Mean	SD	Mean	SD
15-30	30.72	1.42	31.15	0.85
31-45	30.91	0.86	31.26	1.42
46-60	30.00	1.72	30.79	1.00

SD: Standard Deviation

In each age group, the mean olfactory scores of women were higher than men. With increasing age, the scores were decreased.

The designed test is cheap and available and significantly will reduce our need to expensive foreign kits. Perhaps it would be better saying this is a large scale pilot study that must be tested furthermore in other studies and trials.
